# Generating explainable hypotheses for drug repurposing with graph neural networks

**DOI:** 10.1038/s41598-026-50149-2

**Published:** 2026-04-24

**Authors:** Pablo Perdomo-Quinteiro, Emre Guney, Alberto Belmonte-Hernández

**Affiliations:** 1 SSR Department, Universidad Politécnica de Madrid, Madrid, 28040 Spain; 2 STALICLA, Discovery and Data Science Unit, Barcelona, 08039 Spain

**Keywords:** Computational biology and bioinformatics, Drug discovery

## Abstract

Biomedical knowledge discovery increasingly relies on computational tools to uncover patterns in complex datasets, yet generating explainable, evidence-based hypotheses about biological interactions remains challenging. This study introduces *XAIPath*, an interpretable pipeline that leverages biomedical knowledge graphs and Graph Neural Networks (GNNs) to uncover and explain novel drug–disease relationships, aiding applications such as drug repurposing. The pipeline combines GNN-based predictions with a post-hoc interpretability layer that extracts simple paths connecting drug and disease nodes in a biomedical knowledge graph and compares them using MinHash-based similarity. Similar paths are grouped via K-means clustering to build interpretable clusters that represent alternative mechanistic hypotheses. The method was applied to the NeDRex knowledge graph for drug indication prediction, with performance evaluated using AUROC, AUPRC, precision, sensitivity, and specificity. XAIPath achieved strong predictive performance, with AUROC exceeding 95% and AUPRC over 90% across training, validation, and test sets, while precision, sensitivity, and specificity all surpassed 85%. Most high-scoring predictions were supported by existing literature, and the extracted path clusters closely aligned with DrugMechDB annotations, supporting the plausibility of the generated hypotheses. Overall, XAIPath offers a scalable and explainable approach for identifying drug–disease associations, facilitating hypothesis generation and biological validation, and highlighting the value of explainable AI in biomedical research and drug repurposing.

## Introduction

Knowledge discovery– the systematic extraction of clear, novel insight from heterogeneous, often voluminous data sources– has become a cornerstone of contemporary biomedical research^1^. By mining clinical records, omics repositories and real-world evidence at scale, researchers can generate mechanistic hypotheses and therapeutic leads far more rapidly than through intuition-driven experimentation alone.

However, despite advancements in technology and increased investment in research and development, the time and cost required to bring a new drug to market continue to rise.. This is clearly seen with Eroom’s Law^2^, which shows that the number of drugs approved each year per billion of dollars expend is halving every nine years. Despite advancements in technology and increased investment in research and development, the time and cost required to bring a new drug to market continue to rise. This inefficiency has prompted the pharmaceutical industry to seek alternative strategies to develop treatments more quickly and cost-effectively. One of such approaches is drug repurposing, where instead of investing time and resources in creating new drugs, efforts are placed in finding new therapeutic applications to already approved drugs. This process offers a significant head start, as it makes use of existing safety and efficacy data, reducing the time and cost associated with drug development^3,4^.

**Fig. 1:**
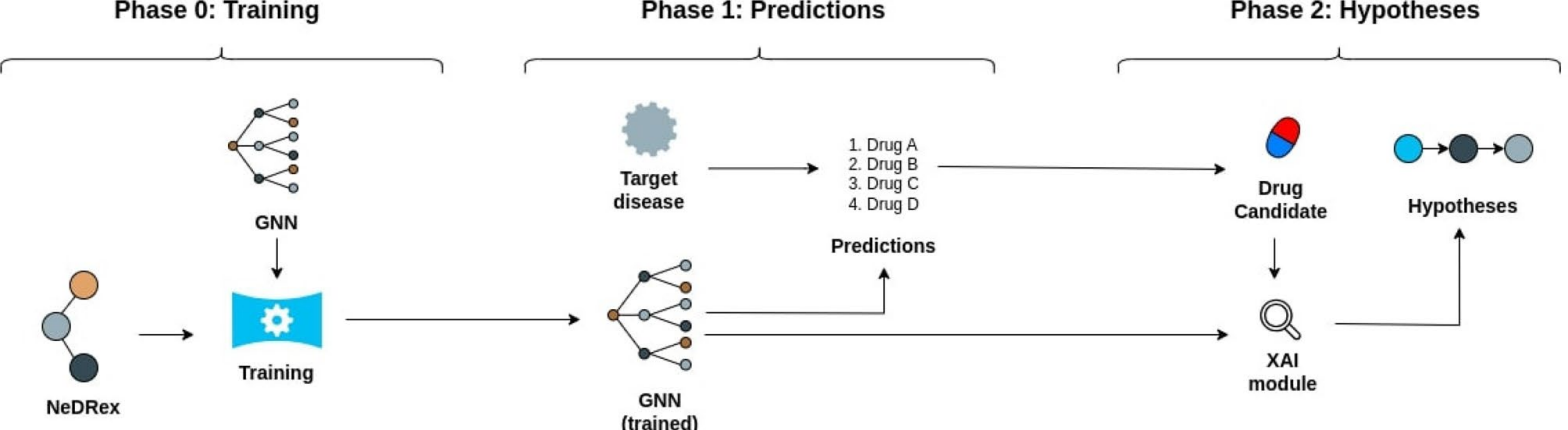
Initial pipeline followed by the proposed XAI method to obtain predictions and hypotheses. In Phase 0, a GNN model (GraphSAGE) is trained to obtain drug predictions. The user can then introduce a target disease and the GNN will propose different potential drug candidates. Next, by providing a drug and a disease the XAI module will generate different potential hypotheses to support the prediction.

Among the drug repurposing methods, one that have gained popularity over the years is Knowledge Graph based drug repurposing^5–8^. A Knowledge Graph (KG) is a data structure that is composed of several nodes, that represent real world entities; and edges, that connect different nodes together. This nodes and edges are usually of different types. For example, in a biological KG one might have *proteins, genes* or *drugs* as node types, and *interacts with, activates, inhibits* as edge types. Some popular biological KGs include: NeDRex^9^, OREGANO^10^, PrimeKG^11^ and PharMeBINet^12^.

There are numerous tools and methods available for analyzing KGs, ranging from traditional graph traversal techniques to AI-based approaches. These latter methods offer advanced capabilities for uncovering complex patterns, predicting relationships, and generating insights that are difficult to achieve with conventional techniques. Among these AI-driven tools, we find Graph Neural Networks (GNNs), which adapt deep learning techniques to graph-structured data. GNNs are particularly effective at capturing both the local and global structure of Knowledge Graphs, enabling them to learn complex relationships between entities. This allows for improved performance in tasks such as node classification, link prediction, and graph embeddings^13^. Additionally, GNNs can generalize across different domains, making them versatile tools for a wide range of applications, from drug repurposing in biomedical research^14,15^ and supporting diagnosis in healthcare^16^ to commercial product recommendation systems^17^. In a biomedical context, previous work has shown that knowledge graphs can successfully support the prediction of adverse drug reactions and pharmacological associations by integrating heterogeneous biomedical data sources and applying machine learning models on the resulting networks^18,19^.

Several GNN models have emerged in recent years. Graph Convolutional Networks^20^ can efficiently aggregate information from neighboring nodes using convolution operations, making them well-suited for tasks like node classification. GraphSAGE^21^ introduces an inductive learning approach, enabling the model to generalize to unseen nodes by sampling and aggregating information from neighbors, which is ideal for handling large, dynamic graphs. Meanwhile, Graph Attention Networks^22^ utilize an attention mechanism to assign different weights to neighbors based on their relevance, allowing the model to focus on the most important nodes and capture complex, heterogeneous relationships. Similarly, we find ULTRA, which learns universal graph representations capable of generalizing across previously unseen knowledge graphs^23^.

While obtaining accurate predictions from GNN models is crucial, it is equally important to ensure that these predictions are transparent and trustworthy, particularly in sensitive areas such as drug repurposing. In this context, understanding the reasoning behind a model’s predictions becomes essential for building confidence in its outputs. This is where Explainable AI (XAI) comes into play, providing methods and techniques to interpret and explain the decisions made by AI models. By making predictions more interpretable, XAI enhances the trustworthiness of AI systems, allowing researchers and clinicians to better understand and validate the underlying mechanisms that support the model’s recommendations.

Several XAI methods have been developed specifically for GNNs to enhance the interpretability of their predictions^24–26^. One notable method is GNNExplainer^27^, which identifies the most influential subgraphs and features that contribute to a model’s predictions, providing insights into the reasoning behind specific outcomes. Another approach, PGExplainer^28^, extends this idea by employing a probabilistic framework to generate explanations based on the relationships among nodes, allowing for a more comprehensive understanding of the graph’s structure and its impact on predictions. Additionally, SubgraphX^29^ focuses on generating interpretable subgraph explanations by leveraging local structures within the graph, highlighting the connections that are most relevant to the predictions made by the GNN. These methods collectively enhance the transparency of GNN models, making them more trustworthy for applications such as drug repurposing.

The primary challenge with existing XAI methods is that the explanations they generate can often be too complex or difficult to interpret, particularly in the context of drug repurposing. On the other hand, more recent attempts aim to overcome this complexity by focusing on drug-pathway-disease triplets, i.e., biological processes connecting drugs and diseases^30^, oversimplifying the mechanisms involved in drug action. In this paper, we propose XAIPath, a GNN-based pipeline that identifies novel relationships between entities in the KG such as drugs and diseases with supporting evidence in terms of simple paths in the KG. In contrast to previous XAI approaches that are either too complex or simplistic, XAIPath is particularly designed to produce drug repurposing candidates and supporting hypotheses that enhance the interpretability of predictions. These hypotheses are constructed as simple paths, making them more accessible and easier for researchers to understand and analyze. By simplifying the representation of relationships between drugs and diseases, our approach aims to facilitate the validation and application of AI-driven insights in a practical research setting. Additionally, while other applications typically validate their approach using a single disease, we make use of DrugMechDB^31^ – a database of curated explanations – to systematically validate our approach.

While we demonstrate our pipeline on the task of predicting novel drug–disease associations, many other relationship-prediction tasks can benefit from the same approach. For instance, relationships such as drug–drug interactions, drug–side effect links, or gene–disease associations can be modeled using KGs and GNNs in a similar fashion. By taking a general GNN-based link-prediction framework our pipeline aims to be broadly applicable to various domains, serving as a flexible tool to extract interpretable hypotheses from biomedical data.

**Fig. 2:**
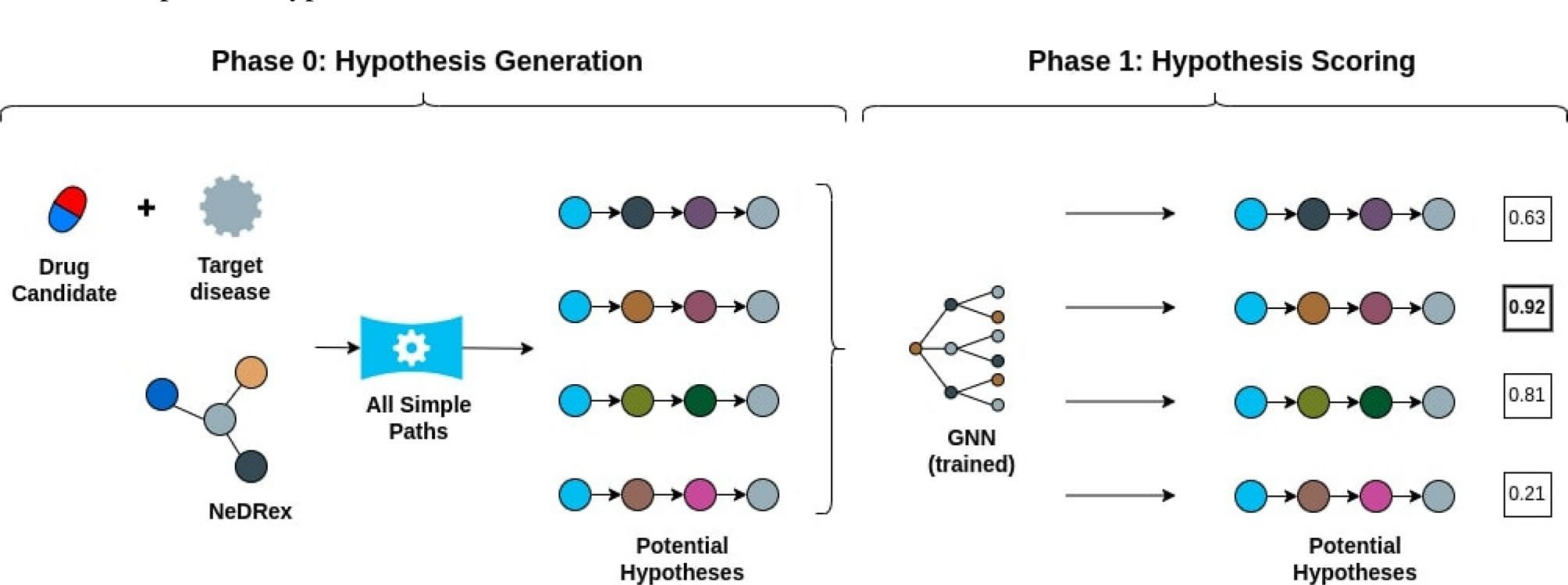
Pipeline based on exhaustive search in the XAI module. After providing a target drug and disease, the XAI module will search for all simple paths connecting drug and disease. This simple paths will serve as input for the GNN, which will assign different scores to the subgraphs. The subgraph with the highest score is considered the best hypothesis.

**Fig. 3:**
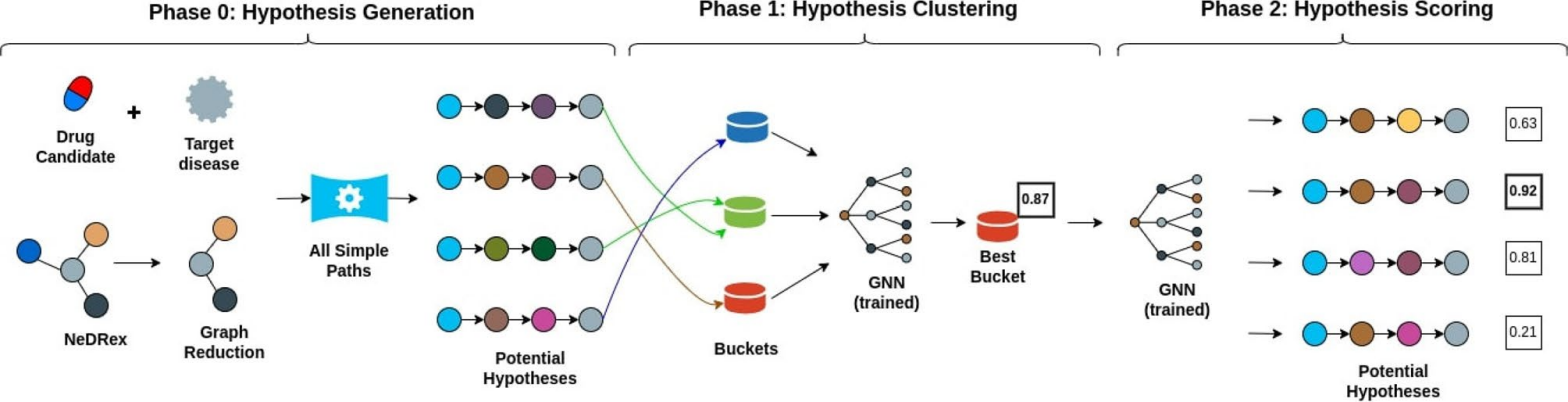
Modified pipeline using reduced KG and clustering in the XAI module. Given a target drug and disease, the XAI module identifies all simple paths in a reduced knowledge graph (KG). These paths are clustered using MinHash and K-Means, and each cluster is scored. The highest-scoring cluster is analyzed in detail, and its top-ranked subgraph is selected as the best hypothesis.

## Methods

The methodology employed in this study is illustrated in Fig. 1. The pipeline consists of two primary components: a prediction segment and an XAI segment. In the prediction phase, a GNN is trained using a link-prediction framework to identify potential drug candidates for specific diseases. The XAI phase begins by identifying all simple paths connecting a drug to a disease. These paths are then scored by the trained GNN, resulting in a ranked list of hypotheses. The entire pipeline has been implemented in Python, utilizing *Networkx* (version 3.3)^32^ and *Pytorch Geometric* (version 0.4.0)^33^.

### Data sources

The data used in this study was extracted from NeDRex^9^, a KG database containing extensive information on proteins, genes, drugs, and their relationships, supporting biological network exploration for purposes such as drug repurposing and disease module identification. Currently, a second version of the NeDRex graph is under development, incorporating new nodes and edges (e.g., side effects, phenotypes, GO terms...). The pipeline was initially developed using the first version but was later switched to the second version. However, after conducting several analyses, we discovered that some nodes and edges present in version 1 were missing in version 2. To address this issue, we employed a KG that combines both versions. To combine both versions, we performed a controlled merge in which newly introduced nodes and edges from version 2 were added to version 1. This approach allows us to incorporate the new nodes and edges introduced in version 2 while minimizing potential errors associated with the unreleased version.

This final version of the NeDRex used in this study contains 413,212 nodes, 8,778,843 edges, 10 node types and 18 edge types. The data was divided in the following way: 80% training, 10% validation and 10% test, as the 80/20 split has been shown to obtain the best performance^34^. The split function provided by PyTorch Geometric ensures that there is no data leakage by assigning reverse edges consistently to the same split, preventing a triplet (*h*, *r*, *t*) and its inverse (*t*, *r*, *h*) from appearing across training, validation, and test sets.

### Relationship prediction using GNN

To maximize the information provided by the NeDRex KG, we used BERT^35^ to generate node embeddings from the descriptions. These descriptions correspond to textual annotations provided in NeDRex for each node entity (e.g., disease summaries, gene or protein functional descriptions, and drug annotations), which contain short, curated explanatory texts. In this case we used “bert-base-uncased” extracted from the *transformers* library. Once more, other LLMs can be incorporated to generate this node embeddings.

This pipeline makes use of the GraphSAGE model^21^ although it can be extended to any GNN model. Specifically, the heterogeneous variant of GraphSAGE was employed to account for the multi-relational structure of the KG. As stated above the data used to train the GNN was the NeDRex graph (combination of versions 1 and 2). The GNN was trained to predict whether a drug and a disease are connected through an edge of type “has indication”. The architecture used was a 3-layer GNN, with an input size of 768 (which corresponds to the embedding output size of the BERT model), a hidden size of 128, and an output size of 64. “SAGEConv” function in Pytorch Geommetric package was used with default parameters to create the layers and ReLU activation function was used between the layers.

The model is then trained to generate an embedding for each node in such way that the dot product of a drug-disease pair will be a score reflecting the probability of a link existing between the drug and the disease. This way, by obtaining and ranking all drug-disease pairs for a given disease, one can obtain the drugs that have the highest possibility of treating the disease. To train the model, both positive and negative samples are required. Positive samples correspond to existing links in the KG, while negative samples are generated synthetically through negative sampling, which creates drug-disease pairs that do not appear in the graph. In our case, one negative sample is created per positive example.

### XAI method

The XAI method proposed in this paper is based on the idea of Mutual Information, already used by other approaches such as GNNExplainer^27^ or PGExplainer^28^, which is given by the formula:

1 $$\begin{aligned} MI(Y, (G_{s}, X_{s})) = H(Y) - H(Y|(G = G_{s}, X = X_{s})) \end{aligned}$$

Here, *H*(*Y*) is the entropy of the original predictions, and $H(Y|(G = G_{s}, X = X_{s}))$ is the entropy of the predictions using the subgraph. However, while other methods make use of optimization to find the best subgraph, our approach starts by generating a set of subgraphs that are then assign a score and ranked. The reason for this methodology is that we want to obtain subgraphs that can be easily understood and interpreted by humans. To do so, all the subgraphs analyzed are in the form of simple paths. A simple path is a sequence of distinct nodes connected by edges, where each node appears only once. In this context, the simple path starts at the disease and ends at the drug, providing a potential explanation that shows why both elements should be connected.

The method is divided into two main steps: obtaining the simple paths and scoring them. There are various algorithms available to find all simple paths between two nodes in a graph^36,37^. For this paper, we employed a modified depth-first search (DFS) algorithm to generate the paths, which is implemented in *NetworkX*^32^. This implementation includes a cutoff parameter that limits the size of the explanations produced. By constraining the size of the explanations, we can enhance the speed of the process while generating simpler and more interpretable hypotheses. It is worth mentioning that the speed increase is not trivial, as increasing the explanation size can exponentially increase the computational time of the process (this will be further explored in the “Results” section). Like many other modules in this pipeline, this method is modular and can be replaced with any other search algorithm; therefore, if a more efficient method becomes available in the future, it can be easily integrated into the workflow.

The second part of the method involves ranking the explanations. As stated above, we aim to identify the subgraph that maximizes the mutual information (MI). The term *H*(*Y*) remains fixed as it represents the entropy computed using the entire graph. Consequently, the problem can be reformulated as minimizing the second term, i.e., finding the subgraph with the lowest conditional entropy. Given that we are investigating a potential drug-disease link (a link to which our GNN has assigned a high score), the subgraph with the lowest entropy is expected to assign the highest score to this drug-disease pair. In other words, the optimal subgraph will provide the most confident explanation for the predicted link. Consequently, to rank the list of subgraphs obtained in the previous step, for a given drug-disease pair, the GNN model will use the subgraph as input (instead of the whole KG) and assign a score to the prediction. The subgraph with the highest score will be the one that contains the most significant nodes and edges for our GNN. In other words, although the GNN is trained to predict individual triples, we interpret each candidate subgraph (cluster) as a constrained version of the original knowledge graph and compute the triple score under this restricted structural context. Thus, the cluster score is defined as the model’s predicted score for the specific drug–disease pair when inference is performed using only the nodes and edges contained in that subgraph. This allows the triple-level predictor to induce a well-defined ranking over clusters, where higher scores indicate subgraphs that better preserve the predictive signal for the target link. A summary of the pipeline, evaluating all potential paths exhaustively can be seen in Fig. 2.

This process represents the most straightforward and direct approach to address the problem. However, as previously mentioned, increasing the length of the explanations leads to an exponential growth in the time required to find all simple paths. To mitigate this, we developed a modified version of the pipeline incorporating two strategies aimed at improving the computational efficiency of the process. The two main components of the hypothesis generation are obtaining all simple paths and scoring these paths, with the former being the most time-consuming step. To address this, we propose a reduction of the size of the KG. Instead of using every node from the original KG, nodes with a large number of neighbors (more than 1000 neighbors) are removed. These nodes greatly increase the complexity of the problem and they tend to be less informative^38,39^. Through out this paper we will refer to this KG as the reduced KG.

The next step involves scoring the generated list of explanations. To accelerate this process, similar explanations are grouped into clusters, which are used as input for the trained GNN which will score each cluster and the explanations within the highest-scoring cluster are further analyzed. Finally, The top-scoring explanation is ultimately selected as the best hypothesis. This approach allows us to significantly reduce the number of explanations that need to be evaluated, with potential speedups proportional to the number of clusters (e.g., clustering into 10 groups could result in a tenfold increase in efficiency, assuming an even distribution of explanations). To effectively group similar explanations, we make use of the MinHash^40^ and K-Means^41^ algorithms, which are widely used in document clustering. The idea of applying this clustering algorithm in the XAI field has already been explored in SAFRAN^42^, which is a rule-based XAI method that used MinHash and LSH^43^ to aggregate similar rules together. The MinHash algorithm generates multiple signatures of size *s* for each explanation, which approximate the Jaccard similarity between pairs of explanations. This ensures that explanations containing similar elements will have similar signatures. The K-means algorithm facilitates the grouping of explanations with a high similarity. In this work, we used signatures of size *s = 128* and a number of clusters equal to *k *= $\sqrt{n}$, where *n* is the total number of simple paths. The result is a set of clusters, each containing a similar number of explanations in them. This workflow is summarized in Fig. 3.

### Evaluation

We evaluated the results using multiple approaches, beginning with an assessment of the GNN’s predictions. Initially, the performance of the GNN is calculated by measuring the AUROC and the AUPRC in the test set. Both of these metrics are a standard when evaluating GNNs in a drug repurposing context^44–46^. Next, a total of 15 diverse diseases were selected, covering a range of categories such as metabolic, infectious, immunologic, and rare diseases. For each disease, the top 10 drugs with the highest scores predicted by the GNN were chosen for further analysis. Notably, the selected drug-disease pairs were not already present in the NeDRex graph, ensuring the novelty of the predictions. A subsequent literature review was conducted to determine whether the identified drug-disease associations had been previously reported.

The evaluation of the generated hypotheses was conducted using the DrugMechDB dataset^31^, which contains manually curated explanations for various drug-disease pairs. To assess the performance of our model, we compared the generated explanations with those provided in DrugMechDB. While DrugMechDB includes 4,846 explanations, our experiments focused on a subset of 464 explanations with a maximum length of four nodes. Due to the absence of certain links in the NeDRex graph, some of the DrugMechDB explanations were adapted to align with NeDRex standards. The dataset is available at: https://github.com/SuLab/DrugMechDB.

To maximize the compatibility of DrugMechDB explanations with NeDRex, we followed a systematic approach. First, we excluded explanations containing more than five nodes to focus on small- to medium-sized hypotheses. Next, we retained only explanations where all nodes existed in NeDRex. Additionally, DrugMechDB explanations often included direct links between proteins and diseases, whereas NeDRex establishes these connections through genes. To resolve this, we manually inserted gene nodes into the DrugMechDB explanations preserving integrity and accuracy of the mechanisms. A similar issue arose with InterPro nodes, which are directly linked to diseases in DrugMechDB but connected through intermediary gene nodes in NeDRex. These nodes were substituted with corresponding entities accordingly. In some cases, conflicts arose from nodes with links absent in NeDRex, such as anatomical structures or biological processes. When substitution was not feasible, we removed these nodes, ensuring the overall explainability of the explanations remained intact. This procedure ensured that only auxiliary or non-mappable entities (i.e., anatomical structures or high-level biological processes) were removed, while preserving the core mechanistic components of each explanation (genes, proteins, and drugs). Although certain higher-level functional annotations (e.g., biological processes) were omitted from the graph representation, these can still be manually consulted by researchers using external databases like Uniprot.^47^

In total, the XAI method was applied to 341 explanations, 82 of which were present in NeDRex. When using the reduced version of the KG combined with clustering, some explanations were missed: either because the nodes involved were absent from the reduced KG or because they were assigned to low-scoring clusters and were not evaluated. This limitation reflects the trade-off between improving computational efficiency and retaining completeness in the explanation process. The evaluation metrics employed to assess the model’s performance were Hits@1, Hits@3, Hits@5 and Hits@10, which measure how well the algorithm ranks the correct explanation^48^. These metrics operate as follows: the model outputs a ranked list of hypotheses, one of which is the known true explanation. The Hits@k metric indicates the proportion of instances where the correct explanation appears within the top k ranked hypotheses.

**Table 1:** Performance evaluation of the GNN across different dataset splits based on AUROC and AUPRC metrics for the training, validation, and test sets.

Dataset Split	AUROC	AUPRC
Training set	0.9501	0.9115
Validation set	0.9532	0.9009
Test set	0.9629	0.9163

**Table 2:** Results of the manual evaluation of the predictions obtained using the GNN.

Disease	Existing	No reported	Potential
Diabetes	7	2	1
Hypertension	6	3	1
Malaria	8	0	2
Tuberculosis	3	6	1
AIDS	0	8	1
DMD	1	8	1
Stroke	3	6	1
Pneumonia	0	8	2
Cystic Fibrosis	0	7	3
Alzheimer	6	2	2
Parkinson	6	3	1
Hemophilia	0	7	3
Influenza	0	8	2
Polycystic kidney	1	7	2
Celiac disease	0	9	1
Total	3.13	5.13	1.18

For each disease, the top-10 predicted drugs were analyzed. The “Existing Association” column shows the number of drugs with known associations for treating the disease; the “No reported Association” column shows the number of drugs with no association in the literature; and the “Potential Association” column indicates the number of drugs with ambiguous or potential associations based on the predictions.

## Results

In this section, we present the results of a series of experiments conducted to evaluate the performance of XAIPath pipeline. The evaluation focuses on the predictions generated by the trained GNN and assesses the quality of the hypotheses formulated during the experiments.

### XAIPath predicts known drug-disease associations accurately

The performance of the GNN model is summarized in Table 1, which presents the evaluation metrics, including the Area Under the Receiver Operating Characteristic Curve (AUROC) and the Area Under the Precision-Recall Curve (AUPRC). Both metrics have been widely used to assess the performance of link prediction problems^49–51^. The model is able to demonstrate an AUROC ($>95\%$) and AUPRC ($>90\%$) across training, validation, and test sets. In addition to these quantitative metrics, we conducted a manual evaluation of 15 diseases by verifying whether the predicted drug-disease associations had been previously reported in the literature. We categorized the associations into three types: (1) Existing Association, where a drug-disease link has been documented; (2) No Reported Association, where no evidence of an association exists in the literature; and (3) Potential Association, where the evidence is inconclusive or limited, such as studies that are unclear or based on animal models. The results of this manual evaluation are presented in Table 2.

### XAIPath generates plausible mechanism-of-action hypotheses

The evaluation of the hypotheses was conducted using the DrugMechDB database. The performance results are summarized in Table 3. When removing nodes from the graph and clustering explanations some of the paths in the KG are lost, either because the node is removed from the KG or because the path falls in a cluster that is not being evaluated. The number of paths used for evaluation is seeing in the “Example” column. The baseline model obtains a Hits@1 score of 0.52, indicating that the explanation with the highest score proposed by the model corresponds to a curated hypothesis. Making use of the reduced version of the KG leads to a slight increase in the Hits@k scores and the computational time. Finally, When applying the boosted model (with the reduced version of the KG and the clustering algorithm), 18 explanations are missed; however, it comes with an increase in the Hits@k scores (achieving a Hits@1 score of 0.71) and being almost 4 times faster than the baseline method. However, this increase could be a product of surviving cases, since some explanations are not evaluated after clustering. For this reason, we performed the same experiment using the same subset of examples. This shows that the increase in Hits@k scores is due to the improved ranking of explanations produced by the clustering-based filtering rather than to differences in the evaluation set.

**Table 3:** Performance of different approaches on hypothesis generation: baseline model, removing high degree nodes from the KG (more than 1,000 neighbors) and removing high degree nodes and clustering explanations.

Approach	Time (s)	Examples	Hits@1	Hits@5	Hits@10	Hits@1*	Hits@10*
Baseline	38190.74	82	0.52	0.60	0.67	0.7	0.78
Remove high-degree	29678.16	80	0.54	0.59	0.66	0.7	0.77
Remove + Minhash + KMeans	10230.95	62	0.71	0.79	0.79	0.71	0.79

We report processing time, number of examples, and Hits@K metrics. * identifies the scores obtained in the same subset through different approaches.

**Table 4:** Table showing the computational time required to generate hypotheses as their maximum size increases.

Path length	Time (Find + Score)	N paths
3	25s (25 + 0)	1
4	27s (26 + 1)	33
5	349s (339 + 10)	289
6	159462s (125982 + 33480)	892956

The time is divided into finding time (to obtain all simple paths) and scoring time (to evaluate each path). The total number of paths is listed in the “N Paths” column. These experiments were conducted using the “Diabetes-Albiglutide” link.

**Table 5:** Table showing the computational time required to run the boosted method with the reduced version of the KG and the clustering algorithm for different explanation sizes.

Path length	Time (Find + Score)	N clusters	N paths in cluster
3	16s (16 + 0)	1	1
4	16s (16 + 0)	5	5
5	23s (23 + 0)	16	19
6	2646s (2591 + 55)	616	783

Additionally, it is also included the number of clusters that are created in each instance and the number of paths in the cluster with the highest score. These experiments were conducted using the “Diabetes-Albiglutide” link.

To assess the efficiency of the algorithm, we conducted a series of experiments to evaluate its performance as the length of the explanation increases. For these experiments, we focused on the Diabetes–Albiglutide pair, as it represents the drug candidate with the highest score predicted by the GNN. The baseline performance of the model is presented in Table 4. This baseline approach involves running the algorithm to identify all simple paths between the drug and the disease, followed by scoring each path. As expected, the number of simple paths grows exponentially with the length of the hypotheses, significantly increasing computational demand. These experiments were carried out using a computer with the following specifications: Intel®Core$^\textrm{TM}$ i9-10900 CPU @ 2.80GHz $\times$ 20, NVIDIA GeForce RTX 3090Ti and 32 GB RAM.

A subsequent experiment was conducted using the boosted approach, which incorporates a reduced version of the KG and clustering algorithms. The results of this experiment is presented in Table 5. It is seen that the new method offers an increase in the computation time, being this specially remarkable in the hypothesis of size 6, where it can be 60 times faster. The main reason for this increase is that, instead of having to score each path, the method just needs to score *k* + *m* points, where *k* corresponds to the number of clusters and *m* corresponds to the number of the number of paths in the highest scoring cluster. This experiment required increased RAM, so an alternative system was used to ensure fair comparison of scoring times between the two approaches. In this case the specifications of the system where: Intel(R) Core(TM) i9-7900x CPU @ 3.30GHz, NVIDIA GeForce RTX 2080 Ti (12 GB) and 64GB of RAM.

Nonetheless, to further assess the consistency of the results, we designed two experiments. In the first experiment, we randomly removed 10% of the nodes from the knowledge graph and compared the resulting explanations to those obtained using the full graph. To avoid removing nodes directly involved in the evaluation, we did not remove drug or disease nodes. We evaluated 100 drug–disease pairs. For each pair, we checked whether the top-ranked explanation generated with the full graph matched the top-ranked explanation produced with the reduced graph. When the explanations differed, we quantified the discrepancy using the Jaccard similarity between the node sets in both explanations.

In addition, we computed a second Jaccard similarity based on node types rather than specific node identities. This type-level comparison treats explanations as equivalent when substitutions occur within the same semantic class (e.g., one gene replaced by another gene), capturing stability at the level of biological roles rather than exact entities. We repeated this procedure using 5-fold cross-validation, generating five different reduced graphs (i.e., different random node removals). The results for each fold are reported in Table 6.

**Table 6:** Table showing the amount of unchanged explanations as well as the similarity between the original graph and the modified graph (reduced number of nodes)

Fold	Unchanged explanations	Jaccard similarity	Jaccard similarity by type
1	100	-	-
2	99	0.33	1
3	99	0.66	1
4	97	0.51	0.83
5	96	0.46	0.66
Total	0.48	0.48	0.87

**Table 7:** Table showing the amount of unchanged explanations as well as the similarity between the original graph and the modified graph (edge permutation)

Fold	Unchanged explanations	Jaccard similarity	Jaccard similarity by type
1	66	0.51	0.94
2	63	0.43	0.92
3	60	0.48	0.96
4	61	0.47	0.95
5	64	0.50	0.92
Total	62.8	0.48	0.94

Similarly, we performed an analysis in which the graph structure was perturbed by modifying its edges. Specifically, we removed 10% of the edges and added the same number of new edges. To avoid directly affecting the evaluation targets, we did not remove edges incident to drug nodes. The same 100 drug–disease pairs were evaluated using a 5-fold cross-validation protocol. As before, explanations generated from the perturbed graphs were compared with those obtained from the original graph using both node-level and type-level Jaccard similarity. The results for each fold are reported in Table 7.

To further assess the importance of the extracted explanatory paths for the model’s predictions, we conducted an additional experiment based on path ablation. While the previous experiments evaluate the robustness of the explanations under graph perturbations, this analysis focuses on their necessity.

We selected a validation set consisting of 3480 positive and negative drug-disease pairs. First, we computed the baseline predictive performance of the model in terms of AUROC. Then, for each prediction, we identified the top-ranked explanatory path and removed all edges belonging to this path from the graph. On average, this corresponds to removing approximately 3 edges per prediction. After this modification, we recomputed the prediction scores and evaluated the resulting AUROC. The results show a decrease in performance from 0.991 to 0.945, corresponding to an approximate 5% drop in AUROC. This indicates that removing only a small number of edges associated with the most relevant explanatory path has a measurable negative impact on the model’s predictions, supporting the claim that these paths capture important predictive structure.

In addition, we conducted a case study focusing on malaria. We analyzed the top 10 predicted drugs for this disease and compared their prediction scores before and after removing the top-ranked explanatory path. We observe a consistent decrease in prediction scores across all considered drugs, with an average reduction of approximately 5.33% (with a maximum drop of 14.12% and a minimun drop of 0.05%). This further supports the relevance of the extracted paths in driving the model’s predictions.

Overall, these results complement the robustness experiments by demonstrating that the extracted explanatory paths are not only stable under perturbations, but also necessary for maintaining predictive performance.

### Example hypotheses generated by XAIPath

**Fig. 4:**
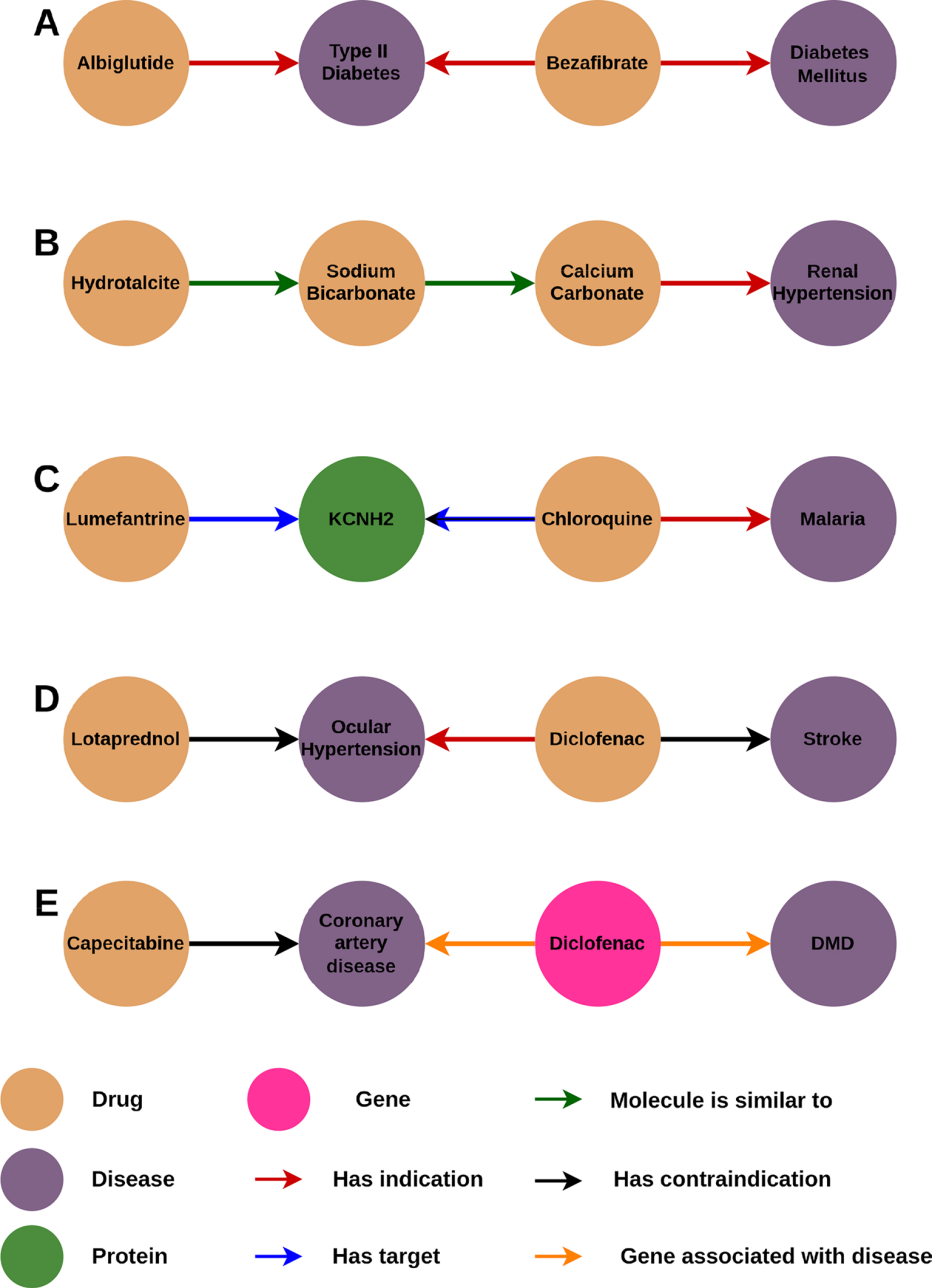
Hypotheses with the highest score for different drug-disease pairs generated by the model. Each subfigure represents the best explanation for the top drug candidate associated with a specific disease, with the maximum hypothesis length set to 4. These hypotheses explore various potential mechanisms linking drugs and diseases: (**A**): Albiglutide-Diabetes Mellitus; (**B**): Hydrotalcite-Renal Hypertension; (**C**): Lumefantrine-Malaria; (**D**): Loteprednol-Stroke; (**E**): Capecitabine-DMD.

Several of the generated hypotheses are illustrated in Fig. 4. Each of these hypotheses represents the best explanation for the top drug candidate for a given disease, with the maximum hypothesis length set to 4. These explanations explore various mechanisms that could support the use of a drug to treat a disease. For instance, Fig. 4A demonstrates that if two drugs treat the same condition and one of them also treats another condition, the other drug may also be effective for that additional condition. Figure 4B illustrates that a drug chemically similar to another drug (based on the Tanimoto coefficient^52^) already approved for a disease may be a suitable candidate for treating the same condition. Similarly, Fig. 4C shows that if a new drug shares a molecular target with an approved drug, it may also be effective in treating the disease. Figure 4D presents the case where drugs with opposing effects on one disease might have opposite effects on another disease. While these hypotheses are not universal rules, they can assist researchers in evaluating whether a drug is a viable candidate for treating a disease. If the model’s hypothesis is ambiguous or lacks biological plausibility, researchers can discard the candidate and consider alternative drugs. An example of this is shown in Fig. 4E, where the hypothesis does not appear to have any biological or logical foundation.

## Discussion

The results highlight the potential of using KGs and GNNs in generating explainable drug repurposing hypotheses. By focusing on simple paths within KGs, we demonstrated that XAIPath could offer clearer, more interpretable hypotheses for the relationships between drugs and diseases. This pipeline, which emphasizes interpretability, is a significant step forward in creating trustable AI systems in the biomedical field, particularly in drug discovery and repurposing.

The GNN model demonstrated strong predictive performance across all dataset splits, as shown in Table 1. The model achieved an AUROC of 0.9501 and an AUPRC of 0.9115 on the training set, with similar results on the validation set (AUROC: 0.9532, AUPRC: 0.9009) and improved performance on the test set (AUROC: 0.9629, AUPRC: 0.9163). These metrics indicate a high level of discrimination and precision in identifying drug-disease associations. Furthermore, a manual evaluation of 15 diseases provided additional qualitative insights into the model’s predictions. As presented in Table 2, for the top 10 predicted drug-disease pairs, 31.3% were found to have an existing association, 51.3% had no reported association in the literature, and 11.8% fell under potential associations with inconclusive or limited evidence. These results suggest the model’s ability not only to accurately identify known drug-disease links but also to propose potential new associations, highlighting its utility in discovering novel therapeutic connections.

A key aspect of the proposed method is its focus on simplicity and human interpretability. Unlike other GNN-based XAI methods, such as GNNExplainer^27^ or PGExplainer^28^, which often generate complex and difficult-to-interpret explanations, our approach generates simple paths that researchers and clinicians can easily validate and understand. This simplicity ensures that the drug candidates suggested by the GNN model are not only plausible but can be logically traced back to known biological mechanisms.

When analyzing the consistency of results, overall, XAIPath proves to be highly consistent under node removal: on average, 98.2% of explanations were identical between the full and reduced graphs. When explanations changed, the mean node-level Jaccard similarity was 0.48, while the mean type-level Jaccard similarity was 0.87. This indicates that differences are typically small (often limited to a single node) and that, even when specific nodes change, they tend to be replaced by nodes of the same type, preserving the explanatory structure. Similarly, under edge permutation on average, 62.8% of explanations remained unchanged. For explanations that differed, the mean node-level Jaccard similarity was 0.48, while the mean type-level Jaccard similarity was 0.94. As expected, the proportion of unchanged explanations is lower than in the node-removal experiment, reflecting the increased difficulty of this setting, which involves both deletion and addition of structural information. Nevertheless, the high Jaccard similarities indicate that the explanatory quality and overall semantic structure are largely preserved despite these perturbations.

The use of clustering techniques, such as MinHash and K-means, for improving computational efficiency without sacrificing the quality of explanations represents a novel contribution. The balance between computational time and interpretability remains one of the biggest challenges in applying GNNs to large KGs, especially when generating subgraphs for complex biomedical data. The boosted approach significantly improves the speed and scalability of the pipeline, making it feasible for large-scale drug repurposing applications.

A key advantage of this pipeline is its modular design, allowing it to be extended to multiple relationship-prediction tasks beyond drug repurposing. For example, by substituting the ‘has indication’ relationship with a ‘causes side effect’ edge, the pipeline could identify novel drug–side effect associations using the same explainable framework. Likewise, switching to a ‘drug–drug interaction’ edge type would enable the detection and ranking of potential drug-drug interacions, along with interpretable path-based mechanisms. Although our current work focuses on NeDRex, the same architecture can be applied to other biomedical knowledge graphs such as PrimeKG, Hetionet, or OREGANO, provided they contain node descriptions or node features compatible with GNN training. Including results from these broader applications remains an exciting avenue for future exploration.

One limitation of our approach is the reliance on existing knowledge within the KG, which may not fully capture emerging data or novel drug-disease relationships. This emphasizes the importance of continuously updating and validating KG databases like NeDRex. Additionally, the performance of the GNN model, while robust, was sometimes limited by the quality of the input data, particularly in cases where drug-disease associations were sparse or incomplete (ie. rare diseases).

Another limitation of our evaluation is the absence of a direct benchmarking comparison against other GNN-specific XAI methods such as GNNExplainer, PGExplainer, or SubgraphX. Direct comparison is challenging because XAIPath is, to the best of our knowledge, the only method that explicitly generates explanations in the form of simple paths, whereas existing approaches are designed to extract subgraphs. We attempted to adapt these methods for comparison; however, their outputs consistently consisted of general subgraphs rather than simple paths, making a structurally fair comparison infeasible. Therefore, the evaluation focuses on assessing XAIPath within its intended explanatory framework rather than against methods optimized for a different explanation format.

Evaluating explanations can many times be a challenging and subjective task, especially in complex domains such as drug repurposing. In this work, we propose the use of DrugMechDB^31^ as an objective alternative to assess how well an XAI model performs in a drug repurposing setting. In contrast to other methods on specific cases studies to perform the evaluation^6,53,54^, DrugMechDB provides a mechanistic basis for evaluation by taking advantage of known drug-disease interactions and biological pathways, thus ensuring that the assessment is based on domain-specific knowledge. The evaluation metrics used, such as Hits@k, demonstrated that the model performs well in ranking potential drug-disease hypotheses, effectively matching its predictions with biologically meaningful mechanisms. This approach not only enhances the reliability of XAI evaluations but also bridges the gap between interpretability and domain relevance in drug discovery. The manual evaluation of the predictions also revealed that some predicted associations, although not currently documented in the literature, are biologically plausible and may warrant further investigation in clinical trials or laboratory settings.

Future efforts will focus on refining the XAI pipeline to improve computational efficiency and scalability. Optimizing clustering techniques, such as MinHash and K-means, or exploring alternative algorithms could further enhance the model’s performance on large and dynamic knowledge graphs. Additionally, integrating richer datasets, including multi-omics data and newly curated drug-disease associations, will expand the KG and improve the model’s ability to generate relevant hypotheses. Although we highlight one particular use case–drug–disease prediction–our choice was driven by data availability and clarity of exposition. Demonstrations of broader applicability to other KG-based biomedical problems, such as DDIs or side effects, would further strengthen the argument that this pipeline functions as a general XAI tool. We leave those expansions for future work, where domain-specific data sets and additional validation metrics can be employed to benchmark performance.

## Data Availability

Additional materials, including details on dataset creation, evaluation data acquisition, methods, and results, are available in the code repository accompanying the paper: https://github.com/PPerdomoQ/XAIPath. The DrugMech dataset used for the evaluation is available in their repository: https://github.com/SuLab/DrugMechDB.
